# Actin Retrograde Flow Regulated by the Wiskott–Aldrich Syndrome Protein Drives the Natural Killer Cell Response

**DOI:** 10.3390/cancers14153756

**Published:** 2022-08-02

**Authors:** Batel Sabag, Moria Levy, Jessica Kivelevitz, Nataly Dashevsky, Aviad Ben-Shmuel, Abhishek Puthenveetil, Fatima Awwad, Mira Barda-Saad

**Affiliations:** The Mina and Everard Goodman Faculty of Life Sciences, Bar-Ilan University, Ramat-Gan 5290002, Israel; sabagba@biu.ac.il (B.S.); morialevy94@gmail.com (M.L.); jesskiv9@gmail.com (J.K.); natashkadash@gmail.com (N.D.); aviad152@hotmail.com (A.B.-S.); raman.abhishek96@gmail.com (A.P.); awwadf183@gmail.com (F.A.)

**Keywords:** ARF, natural killer cells, SHP-1, myosin IIA, WASp, mechanotransduction

## Abstract

**Simple Summary:**

Immune cells sense mechanical stimuli from their microenvironment by transducing them into biochemical signals through mechanosensors, in a process called mechanotransduction. The cytoskeleton mediates mechanotransduction in response to local environmental changes. The cytoskeletal component, actin, provides the structural basis for the NK cell immunological synapse (NKIS) and polarization of the signaling complex and secretory apparatus. Actin-mediated mechanotransduction is essential to adjust and to improve the natural killer (NK) cell response to changes induced by the microenvironment. Wiskott–Aldrich Syndrome protein (WASp), another key cytoskeletal component, plays a role in actin filament nucleation, and hence it’s branching toward the membrane. Here, we found that WASp regulates mechanotransduction by controlling F-actin movement thereby modulating NK cell signaling and the equilibrium between activation versus inhibition. WASp regulation of the actin retrograde flow (ARF) provides a novel mechanism to modulate the functional outcome of NK cells in response to their environment.

**Abstract:**

Understanding the crosstalk between natural killer (NK) cells and the tumor microenvironment (TME) has enhanced the potential of exploiting the interplay between activation and inhibition of NK cells for immunotherapy. This interaction is crucial for understanding how tumor cells escape NK cell immune surveillance. NK cell dysfunction is regulated by two molecular mechanisms, downregulated activating receptor ligand expression on the tumor cells, and upregulated inhibitory signals delivered to NK cells. Recent studies demonstrated the role of mechanotransduction in modulating NK cell responses in the TME. The immunological synapse represents a functional interface between the NK cell and its target, regulated by Actin Retrograde Flow (ARF), which drives the adhesion molecules and receptors toward the central zone of the immunological synapse (IS). Here, we further characterize the role of ARF in controlling the immune response of NK cells, using CRISPR/cas9-mediated Wiskott–Aldrich Syndrome protein (WASp) gene silencing of NK cells. We demonstrate that WASp regulates ARF velocity, affecting the conformation and function of the key NK inhibitory regulator, SH2-domain containing protein tyrosine phosphatase-1 (SHP-1), and consequently, the NK cell response. Our results demonstrate the potential of modulating the biophysical and intracellular regulation of NK activation as a promising approach for improving immunotherapy.

## 1. Introduction 

Natural killer (NK) cells are lymphocytes of the innate immune system, which share some characteristics with adaptive immunity, such as an expansion of pathogen-specific cells, the generation of long-lasting ‘memory’ cells, and the ability to mount an enhanced secondary recall response [1,2]. NK cells are at the forefront of immune response, scanning and killing tumor-transformed or virally infected cells [3]. NK cell activation is controlled by the balance between activating and inhibitory signals transduced upon engagement of varied activating and inhibitory receptors expressed on the NK cell surface [4]. Ligation of the inhibitory receptors to major histocompatibility complex class I (MHC-I) molecules expressed on healthy self-cells leads to the inhibition of NK cell activity [5]. This engagement antagonizes activating pathways through the recruitment of the tyrosine phosphatase Src homology region 2 domain-containing phosphatase-1 (SHP-1), a key protein that is recruited to the NK immunological synapse (NKIS), dephosphorylating key signaling proteins such as VAV1, PLCγ1/2, and LAT, which are crucial for NK cell activation, resulting in an inhibitory response [6,7,8]. Over the last decade, the actin cytoskeleton was shown to play a central role in NKIS formation and maturation, and as a mechanosensor for NK cell responses to mechanical stimuli, beyond its critical role as a scaffold for signaling molecules [9]. One such molecule, the Wiskott-Aldrich syndrome protein (WASp), activates actin polymerization by serving as a nucleation promoting factor (NPF) for the Arp2/3 complex, a macromolecular machinery that nucleates branched actin filaments in response to cellular signals [10,11,12]. Upon NK cell activation, WASp forms a multi-protein complex with WASp-interacting protein (WIP), actin, and myosin, and these associations are abrogated during NK cell inhibition [13,14,15,16]. WASp impacts multiple facets of NK cell activity that depend on cytoskeletal turnover, such as NKIS formation, NK cell migration, and cytotoxicity [17]. Indeed, WASp-deficient cells were shown to form an impaired cytotoxic NKIS, due to a decrease in actin accumulation [18]. Impaired WASp activity also negatively impacts NK cell motility. WASp signaling cascades are essential for LFA-1-mediated migration in response to chemokine receptor-induced inside-out signaling [19]. Interestingly, WASp deficiency was also shown to promote tumor growth in-vivo [20]. Interaction of NK cells from WASp-deficient patients with susceptible target cells results in reduced accumulation of activating receptors and F-actin, and reduced cytolytic granule and perforin secretion at the NKIS [18,21]. These findings support the regulatory role of WASp in cytoskeleton organization, which also affects adhesion molecule activation within the NKIS [22]. A reciprocal regulatory mechanism exists between WASp and actin turnover [23] in which WASp-dependent actin polymerization is responsible for further accumulation of WASp at the NKIS. While the role of WASp in actin polymerization is very well established, its effect on actomyosin retrograde flow (ARF) has not been described in detail. 

ARF describes the continuous centripetal movement of the cell peripheral actin networks. Previously, myosin IIA was shown to play a role along with actin networks at the immune-target cell contact site [24]. Myosin IIA is an ATP-dependent motor molecule that crosslinks actin filaments, and slides over them as they traverse each other, contracting them into actomyosin filament bundles, and thereby establishing the molecular infrastructure for ARF [9,25,26,27]. Myosin IIA has a critical role in immune cells, and it is required for cell motility, migration, and adhesion. It was shown in NK cells that phosphorylation of the myosin IIA tailpiece on Ser 1943 regulates single myosin IIA molecule association with lytic granules to promote NK-cell cytotoxicity [28,29]. In addition, the actomyosin network is able to mediate changes in protein conformation through generation of mechanical force, thereby converting a mechanical stimulus into biochemical signals by a process called mechanotransduction. Accordingly, the ‘pushing’ and ‘pulling’ force generated by actin polymerization and myosin contraction can exert regulatory effects on signaling cascades. Nevertheless, the role played by ARF in NK cell activation has not been widely studied. 

Recently, we demonstrated a direct molecular interaction between β-actin and SHP-1 in NK cells, and we showed the effect of ARF arrest in reducing SHP-1 activity. While fast ARF was observed within the activating NKIS, slow ARF is seen at the inhibitory synapse [9]. Furthermore, we demonstrated that inhibition of ARF leads to SHP-1 inactivation by inducing its auto-inhibited conformational form, resulting in high phosphorylation of the SHP-1 substrate, VAV1 [9]. 

Here, we extend our understanding of the cross talk between the actomyosin network and the SHP-1 signaling cascade and demonstrate a novel regulatory role of WASp and myosin IIA on ARF velocity and SHP-1 activity. Our results might explain the fact that NK cells are generally poorly responsive to solid tumors, as opposed to hematological malignancies, most likely due to the influence of the solid tumor’s stiff microenvironment on NK cell activity [27,30,31,32]. Understanding the mechanisms controlling NK cell cytotoxicity will enable us to develop next generation immunotherapeutic approaches that enhance NK cell anti-tumor activity [32]. 

## 2. Materials and Methods

### 2.1. Cells

The human YTS NK cell line, expressing the inhibitory receptor KIR2DL1 (referred to as YTS-2DL1), and two variants of the 721.221 B-lymphoma cell line, 721.221-HLA-Cw4 (HLA allele serving as a ligand for KIR-mediated inhibitory signals) and 721.221-HLA-Cw7 (irrelevant HLA, enabling NK activation) were generously provided by Prof. Ofer Mandelboim of the Hebrew University, Jerusalem. These cells were routinely tested for mycoplasma: YTS-2DL1 cells were cultured in Iscove’s medium (Biological industries, Cat# 01-058-1A, Is) supplemented with 10% fetal bovine serum (FBS), 2 mM L-glutamine, penicillin (50 μg/mL), streptomycin (50 μg/mL), and 50 μM 2-mercaptoethanol; and 721.221 cells were cultured in RPMI 1640 (Sigma-Aldrich, Saint-Louis, MO, USA) supplemented with 10% FBS, 2 mM L-glutamine, penicillin (50 μg/mL), streptomycin (50 μg/mL), 1% non-essential amino acids, and 1% sodium pyruvate.

Human primary human NK cells (pNKs) were isolated from PBMCs of healthy donors using a EasySep human NK cell enrichment kit (STEM Cell Technologies Vancouver Canada, Cat# 19055); KIR2DL1 expressing pNK (pNKs KIR2DL1^+^) cells were subsequently isolated using anti-KIR2DL1 PE labelled antibody (Milteyni Biotec, Bergisch Gladbach, Germany, Cat# 130-099-029) via magnetic separation using the EasySep human PE isolation kit (STEM Cell Technologies, Cat# 18551). The observed purity was >95%.

### 2.2. Cell Transfection by Electroporation

YTS-2DL1 or pNKs KIR2DL1^+^ cells were transfected with a Lonza Nucleofector™ 2b Device using the manufacturer’s protocol X-001 or U-001, respectively. Transiently transfected cells were used 24–48 h following transfection. Stable clones were derived from transiently transfected cells by cell sorting. Cell sorting was performed using the FACSAria III flow cytometer (Becton Dickinson (BD) Biosciences, Erembodegem, Belgium).

### 2.3. Fluorescence Resonance Energy Transfer (FRET)

Double-color FRET was performed using CFP (excitation wavelength: 468 nm; emission filter, wavelength: 465 to 510 nm) as a donor, and YFP [excitation wavelength: 514 nm; emission filter wavelength: 530 nm long-pass (LP)] as an acceptor. When the fluorescent tags are at a distance of 1–10 nm, CFP excitation at 468 nm causes YFP excitation, with emission measured at 530 nm, indicating FRET efficiency. Dynamic fluorescent and DIC images were collected on a Zeiss laser scanning (LSM) 510 Meta confocal microscope. Measurement and calculation of FRET efficiency were performed as previously described [6].

### 2.4. Confocal Microscopy

Dynamic fluorescent and differential interference contrast microscopy (DIC) was performed, and all images were collected using a Zeiss 510 Meta confocal microscope using a 63× Plan-Apochromat objective (Carl Zeiss, Jena, Germany). The images were extracted using the LSM browser (Carl Zeiss).

#### 2.4.1. Live Cell Microscopy

A Zeiss Observer Z1 equipped with a Plan Apochromat × 100/1.4 NA oil objective was used to obtain live movies. An ORCA-ER digital camera (Hamamatsu Photonics, Herrsching am Ammersee, Germany) was used for imaging with a frame interval of 1 s and an exposure of 500 ms. The cells were maintained at 37 °C in 5% CO_2_ during imaging.

#### 2.4.2. Cellular Imaging of Synapse Intensity

For NK cell–target cell conjugation assays, 5 × 10^5^ Cw4 target cells were seeded over slides in 300 mL of Opti-MEM for 2 h at 37 °C, after which any non-adherent cells were removed by rinsing. Next, 5 × 10^5^ NK cells were seeded over the slides, and allowed to form conjugates with the target cells at 37 °C for 10 min. The cells were then fixed for 30 min with PFA and twice washed with PBS^−^/^−^. Images were collected on a Zeiss LSM 510 Meta confocal microscope.

Phospho-protein accumulation at the NKIS was evaluated by permeabilizing cells with 0.1% Triton X-100 for 5 min followed by blocking in a PFN Buffer (PBS without Ca^+2^ and Mg^+2^ + 10% FCS + 0.02% sodium azide) for 1 h in 2% goat serum (Jackson ImmunoResearch, West Grove, PA, USA). Cells were incubated in a blocking medium buffer with the indicated primary antibodies diluted for 1 h, followed by staining for 30 min with isotype-specific AlexaFluor-conjugated antibody. Cells were washed three times with PFN between steps. The relative fluorescence intensity of the phosphorylated form of the proteins at the IS was determined by measuring the ratio between fluorescence intensity at the NKIS relative to a site on the cell that did not include the NKIS, using ImageJ1.53k Wayne Rasband and contributors—National Institutes of Health, USA.

#### 2.4.3. Image Analysis of F-Actin Dynamics 

Analyses of actin dynamics from live cell videos were performed by preparing kymographs and then tracing the movement of the fluorescently tagged actin filaments. The resulting F-tractin-GFP traces were then used for calculating actin dynamics. The kymographs were processed using the Unsharp Mask filter in ImageJ (with values of radius 5 and mask weight 0.8) in order to simplify the identification of F-actin traces. NKIS radius velocity over time of NK spreading during activation on slide was calculated by measuring the velocity of actin in the NKIS radius. The radius was normalized to one and data points were then split into bins according to their relative cellular location. For each bin, average velocity and standard error were calculated, while noting the time from the beginning of cellular spreading. Data points were then split into bins according to the time index. For each bin, average velocity and standard error were calculated. Image analyses of F-actin dynamics were performed using ImageJ. For data binning, PERL and Microsoft Excel were used. GraphPad Prism software was used for statistical analysis. 

### 2.5. Lysis of NK Cells and Western Blot (WB) 

Cells were centrifuged, supernatant was cleared, and the cells were re-suspended in lysis buffer as previously described [9,33,34]. Cells were placed on ice for 30 min and then centrifuged. The cell lysate was collected and moved to fresh tubes. Sample buffer was then added to the cell lysates and samples were heated for 5 min at 100 °C and then centrifuged at 10,000× *g*. 

Western blots were performed as described [6,9,34]. 

The primary antibodies for WB, PTP, and Imaging were: Rabbit anti-pVAV1 (Y160) (Bio Source Cat# Bs44482, Invitrogen, Thermofisher); human anti-CD28 (BioLegend, London, United Kingdom Cat# 302934); Rabbit anti-SHP-1 (C-19) (Santa-Cruz Biotechnology, Heidelberg, Germany, Cat# sc-287); Mouse anti-GAPDH (0411) (Santa-Cruz Biotechnology, Heidelberg, Germany, Cat# sc-47724); and Mouse anti-WASp (D1) (Santa-Cruz Biotechnology, Heidelberg, Germany, Cat# sc-5300).

Secondary Antibodies were: Goat anti-Rabbit (Santa Cruz Biotechnology, Heidelberg, Germany, Cat# sc-2004) and Goat anti-Mouse (Jackson ImmunoResearch, West Grove, PA, USA, cat# 115-035-003). 

### 2.6. Protein Tyrosine Phosphatase (PTP) Assay

SHP-1 catalytic activity was determined by measuring the hydrolysis of the exogenous substrate, p-Nitrophenyl Phosphate (pNPP) by SHP-1, as previously described [9,35]. 

### 2.7. CRISPR/Cas9 Gene Knockdown

YTS-2DL1 WASp knock out (referred to as WASp KO) cells were generated with CRISPR/CAS9 gene knockdown technology, according to published protocols [36]. The pSpCas9-(BB)-2A-GFP (PX458) vector was purchased from Addgene plasmid # 48138. An online CRISPR design tool (Zhang Lab, https://zlab.bio/guide-design-resources, accessed on 20 July 2022, CRISPOR) was used to construct the WASp locus RNA guide sequences. Lonza Nucleofector 2b, protocol X-001 was used to transfect the YTS-2DL1 cells. A GFP gene marker was introduced into the vector. GFP-positive cells, were obtained by cell sorting using a FACSAria (Becton Dickinson Biosciences) cell sorter 24 h after transfection. After recovery for 1 day, the cells were counted and cloned in 96 well plates at a concentration of 1 cell per well. Following colony growth, individual colonies were screened for WASp expression.

### 2.8. Cytotoxicity Assay/[^35^S] Met Release Assay

NK cytolitic activity was determined by measuring the release of [S^35^] Met into the medium by the lysed target cells as previously described. Briefly, 221-Cw4 or Cw7 Target cells were labeled with 0.2 mCi/mL [S^35^] Met for 12 to 16 h and washed twice. The NK and the target cells were then co-incubated for 5 h in the medium at 37 °C at an effector-to-target ratio of 10:1. The percentage cell lysis were calculated by the following equation: % specific lysis = [(sample signal − spontaneous release)/(maximal release − spontaneous release)] × 100.

### 2.9. CD107a Degranulation Assay

YTS-2DL1 WT or YTS-2DL1 WASp KO cells (2 × 10^5^) were co-incubated with mCherry expressing 221-Cw4 or Cw7 cells (6 × 10^5^) at an effector to target ratio of 1:3 for 2 h at 37 °C in the presence of monensin (2µM) (BioLegend, London, United Kingdom Cat 420701). pNK KIR2DL1^+^ cells were transfected with N.S. siRNA (250 pmol) or WASp siRNA (250 pmol) using Lonza Nucleofector 2b, protocol U-001. The cells were then co-incubated with 221-Cw4 or Cw7 target cells at an effector to target ratio of 1:3 (2 × 10^5^:6 × 10^5^) for 5 h at 37 °C in the presence of monensin (2 µM) (BioLegend). 

YTS-2DL1 or pNKs KIR2DL1^+^ cells were then washed and stained using CD107a FITC antibody at a dilution of 1:25 for 30 min on ice. Cells were washed twice and analyzed by FACS (BD FACS Diva software, NJ, USA). YTS-2DL1 cells were distinguished from the targets via mCherry expression and pNKs KIR2DL1^+^s were distinguished from the targets based on FSC vs. SSC. The analysis was performed using FlowJo v10.8.1. Ashland, OR, USA.

### 2.10. Measurement of Intracellular Calcium (Ca^2+^) Concentration

YTS-2DL1 WT or YTS-2DL1 WASp KO cells (1 × 10^6^) were incubated with RPMI1640 medium containing 5 Mm Indo-1-acetoxymethylester (Teflabs, Interchim Innovations, Montlucon Cedex, France, FP-AM608A) and 0.5 mM probenecid (MPB) at 37 °C for 45 min. The cells were washed twice, resuspended in RPMI 1640 without phenol red and containing 10 mM Hepes and 0.5 mM probenecid, and then maintained at room temperature for 20 min. The cells were incubated at 37 °C for 5 min before measurement, analyzed for 1 min to establish the basal intracellular Ca^2+^ concentration, then mixed at a 1:1 ratio with 221 target cells. Ca^2+^ influx was measured using a two-filter setup within the Synergy H1, Microplate Reader (BioTek, Houston, Texas, USA), Gen5 3.05 software. Filter Set 1 (Ca^2+^ short): Excitation: 340/30, Emission: 400/10. Filter Set 2 (Ca^2+^ long): Excitation: 340/30, Emission: 485/40.

### 2.11. Statistical Analysis

Data calculations, data graphing, and statistical analysis were conducted using Microsoft Excel (v14.7.2, Microsoft Corporation, Washington, DC, USA) and GraphPad Prism 9.0.1 (GraphPad Software, Inc., San Diego, CA, USA). *p* values for two conditions were calculated using a one sample t-test or two-tailed unpaired t test. Where more than two conditions were compared, one-way or two-way ANOVA with a Tukeys’ post hoc-test was used to calculate the *p* values. Data are depicted as means ± SEM. Biological replicates and statistical parameters are described in the figure legends.

## 3. Results

### 3.1. The Role of WASp and Myosin IIA in ARF Dynamics at the Activating NKIS

**Figure 1:**
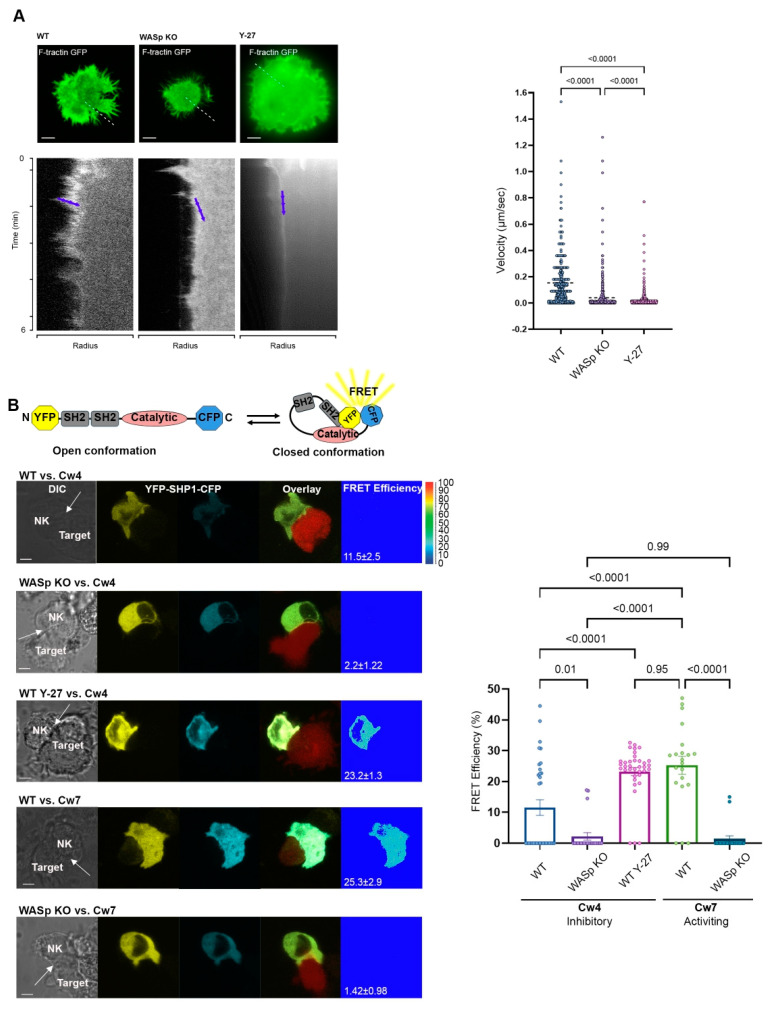
Characterization of F-actin dynamics and SHP-1 conformation. (**A**). Coverslips coated with anti-CD28 were seeded with YTS F-tractin GFP cells and imaged at one frame per second in a single focal plane. Kymographs of F-actin dynamics were compiled along the contact site radius (represented as a white dashed line) and analysis of F-actin traces was compiled into a graph to indicate the F-actin velocity (µm/s) at the contact site (YTS/anti-CD28: 12 movies, YTS treated with Y-27/anti-CD28: m 7 movies, WASp KO/anti-CD28: 13 movies). Representative images from movies are shown, and blue arrowheads indicate traces. Scale bars indicate 5 µm. Data are represented as the mean. Statistical significance was calculated with one way ANOVA test. Movies are provided in the supplementary data. (**B**). Schematic illustration of the YFP-SHP1-CFP FRET sensor. Active “open” SHP-1 conformation results in a large distance between the two fluorescent proteins with no FRET signal. In the inactivated, “closed” SHP-1 conformation, the N- and C-termini are found in close proximity, resulting in high FRET efficiency. YTS-2DL1 cells transiently expressing YFP-SHP1-CFP and 221-Cw4 (inhibitory) or Cw7 (activating) target cells expressing mCherry were co-incubated on slides and were fixed following 5 min of incubation at 37 °C. FRET analysis was performed as described in the Materials and Methods. The representative graph summarizes the mean percentage FRET efficiencies ±SEM (*n* = 33 for Cw4/YTS-2DL1; *n* = 21 for Cw7/YTS-2DL1; *n* = 36 for Cw4/YTS-2DL1 Y-27; *n* = 22 for Cw4/WASp KO; and *n* = 20 for Cw7/WASp KO from three independent experiments). The FRET channels at the NKIS are indicated. Scale bars indicate 5 µm. White arrows indicate the NKIS site formed between YTS and target cells. Statistical significance was calculated with one way ANOVA; the data in the graph represent means ± SEM.

We previously demonstrated that ARF exhibits a different velocity during activating versus inhibitory interactions; the velocity during activation is rapid compared to during inhibition, and it plays an important role in determining the SHP-1 conformational state and enzymatic activity. Moreover, ARF arrest by the pharmacological inhibitor, Jasplakinolide (JAS), during inhibitory conditions leads to SHP-1 inhibition [9]. Furthermore, we demonstrated that F-actin accumulates at the periphery of the activating NKIS. In contrast, myosin IIA is depleted from the lamelliopodia (LP), which is a preferred site for actin dynamics and retrograde flow [9,37,38,39], and accumulates mainly at the lamellum (LM) and the cell body (CB) during formation of both inhibitory and activating NK synapses. Recent studies have shown that F-actin polymerization depends on activation of the Arp2/3 protein complex by multiple nucleation-promoting factors, including WASp, and on contractility generated by myosin IIA [40]. Due to the importance of WASp and myosin IIA in F-actin dynamics, we sought to elucidate their roles in ARF and in regulating SHP-1 activity through their influence on actin polymerization and contractility, respectively. To investigate the role of myosin IIA in ARF regulation and SHP-1 conformation structure and activity, YTS-2DL1 cells expressing F-tractin GFP (Figure 1A and Video S3) were treated with the ROCK inhibitor, Y-27632 (Y-27).

The cells were then seeded over surfaces pre-coated with stimulatory anti-CD28 antibody previously shown activate YTS cell cytotoxicity [41], and the ARF velocity was determined. By measuring the F-actin network at the LP of the activating NKIS, we detected fast and continuous retrograde flow, while in cells treated with the myosin IIA inhibitor, Y-27, this continuous flow was delayed and barely detectable (Figure 1A, Videos S1 and S3). These results show that the myosin IIA inhibitor (Y-27) induced the complete arrest of ARF, indicating that myosin contractile forces regulate F-actin flow in NK cells (YTS-2DL1 Y-27 0.017 ± 0.0005 µm/s).

**Figure 2:**
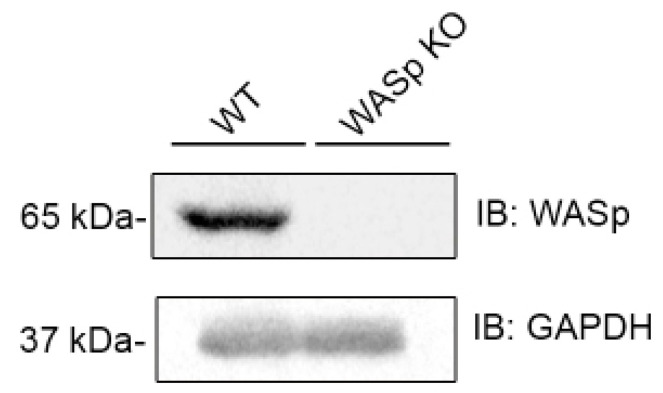
CRISPR Cas9 knockout of WASp in YTS-2DL1 cells. YTS-2DL1 cells and YTS-2DL1 WASp KO were lysed and subjected to western blot with anti-WASp and anti-GAPDH antibodies.

ARF movement is driven by actin polymerization and de-polymerization cycles. It was recently shown that WASp is required for actin accumulation at the site of LFA-1 ligation in NK cells [42]. To determine whether WASp plays a role in ARF dynamics, we prepared a WASp knockout cell line (WASp KO YTS-2DL1) using CRISPR Cas9 technology (Figure 2). 

Imaging of F-actin dynamics was performed at the contact site of fully spread NK cells (Video S2). The ARF velocity of YTS-2DL1 WASp KO expressing F-tractin GFP was quantified using kymograph analysis. F-actin features were monitored at the outer margin of the kymographs, representing the LP (Figure 1A; blue arrows). WASp KO cells had significantly slower ARF movement than YTS-2DL1 cells (expressing WASp WT) (WASp KO 0.04 ± 0.0001 µm/s; WASp WT 0.15 ± 0.0022 µm/s, *p* ≤ 0.0001). These results indicate that ARF movement is highly dependent on WASp. Strikingly, the absence of WASp hampered the rapid ARF movement of the activating synapse, resulting in slower rates of ARF (Figure 1A). 

### 3.2. The Role of WASp in Regulating SHP-1 Conformational State

SHP-1 dephosphorylates signaling molecules following NK cell interactions with inhibitory ligands expressed on the target cell, thereby inhibiting NK cell activity. SHP-1 is found in a closed auto-inhibited conformational state in which its N’-terminal SH2 domain interacts with its catalytic domain. Since we found that the absence of WASp leads to slower ARF of activating NK cells and a myosin IIA inhibitor induced ARF arrest, we next examined whether such attenuation of ARF velocity affects the SHP-1 conformational state. Previously, we demonstrated that moderate actin flow at the NKIS enables SHP-1 to remain in an open active conformation [9]. Thus, we determined whether the slower velocity observed in WASp KO cells switches the SHP-1 conformational state to its “open” active status, similar to that found in a conventional NK inhibitory synapse. To address this question, the YFP-SHP1-CFP Fluorescence Resonance Energy Transfer (FRET) sensor was used [6,9,33]. This SHP-1 FRET sensor was designed with SHP-1 N’ and C’ terminal ends tagged with YFP and CFP, respectively. NK cells expressing YFP-SHP1-CFP were examined following interaction with 221-Cw4 (inhibitory) or 221-Cw7 (activating) target cells expressing mCherry to distinguish between targets and effectors, as previously described [9,33,43]. The intra-molecular proximities of the doubly-tagged SHP-1 were measured by FRET analysis. As expected, the FRET efficiency between SHP-1 N’- and C’-terminus in the WASp KO cells was significantly lower in comparison to WT YTS-2DL1 (Figure 1B; 2.2 ± 1.22%, second panel vs. 11.5 ± 2.5%, first panel; *p* = 0.01). Thus, in WASp KO cells, SHP-1 possesses an open, active conformation in both activating and inhibitory synapses. No changes in the FRET efficiency were detected in the WASp KO cells in response to different target types (Figure 1B; 2.2 ± 1.22%, second panel vs. 1.42 ± 0.98%, fifth panel; *p* = 0.99) indicating that the synapse is sustained in a SHP-1 mediated inhibitory state. Interestingly, treatment with Y-27 under inhibitory conditions led to a significant increase in the FRET efficiency, similar to levels that were observed under activating interactions (Figure 1B; WT Y-27/Cw4 23.2 ± 1.3%, third panel vs. WT/Cw7 25.3 ± 2.9%, fourth panel; *p* = 0.95). Inhibition of myosin IIA leads to ARF arrest and to SHP-1 inactive ‘closed’ conformation. These results suggest that WASp and myosin IIA play a role in determining the SHP-1 conformational state via ARF regulation. 

### 3.3. The Effect of WASp on SHP-1 Activity at the NK Activating and Inhibitory IS

**Figure 3:**
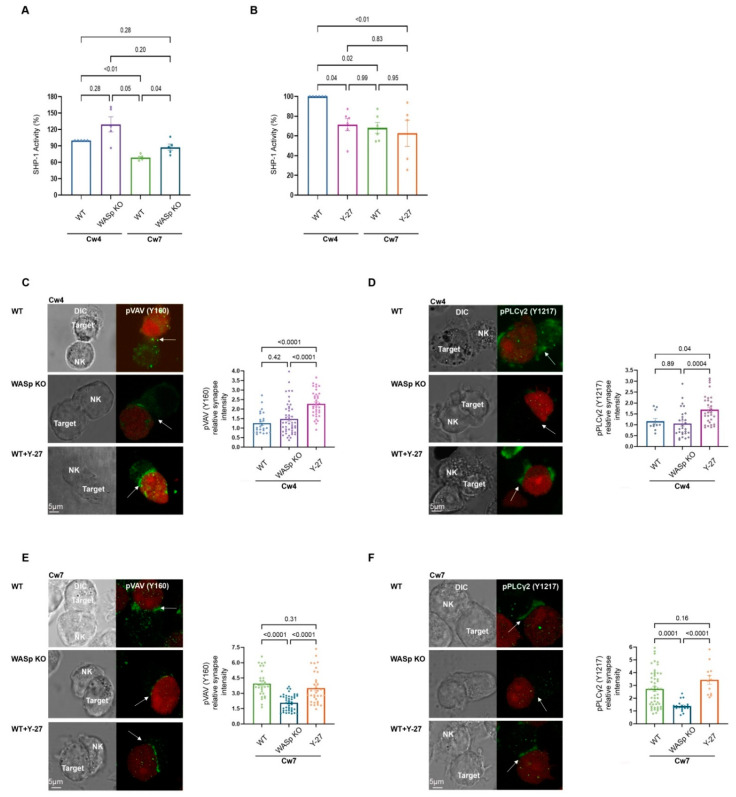
F-actin retrograde flow dictates SHP-1 catalytic activity. (**A**,**B**). YTS-2DL1 and WASp KO cells (**A**), or YTS-2DL1 treated with Y-27 (**B**), and were incubated at 37 °C with 221-Cw4 (inhibitory) or Cw7 (activating) target cells for 5 min, and SHP-1 activity was determined, as detailed in the Materials and Methods. Graphs summarize percentage of relative SHP-1 catalytic activity from five or four independent experiments, respectively. Statistical significance was calculated with one way ANOVA test, the data in the graph are represented as mean ± SEM. (**C**–**F**). YTS-2DL1 untreated, YTS-2DL1 treated with Y-27, or WASp KO cells and mCherry-expressing 221-Cw4 or 221-Cw7 target cells were co-incubated on slides at 37 °C followed by staining with (**C**,**E**) anti-pVAV1 (Y160) or (**D**, **F**) anti-pPLCγ2 (Y1217) and secondary Alexa-488 Ab to determine the pVAV1 and pPLCγ2 accumulation at the NKIS. The effector and target cells were distinguished by the expression levels of mCherry. Scale bars indicate 5 µm. The NKIS site formed between YTS-2DL1 and target cells is indicated by white arrows. (**C**). Representative images and a representative graph summarizing the relative pVAV1 (Y160) synapse fluorescence intensities (*n* = 25 for Cw4/YTS-2DL1; *n* = 36 for Cw4/YTS-2DL1 Y-27; *n* = 47 for Cw4/WASp KO). Statistical significance was calculated with one way ANOVA test, the data in the graph are represented as means ±SEM. (**D**). Representative images and a representative graph summarizing the relative pPLCγ2 (Y1217) synapse fluorescence intensities (*n* = 11 for Cw4/YTS-2DL1; *n* = 34 for Cw4/YTS-2DL1 Y-27; *n* = 29 for Cw4/WASp KO). Statistical significance was calculated using one way ANOVA. (**E**). Representative images and a representative graph summarizing the relative pVAV1 (Y160) synapse fluorescence intensities (*n* = 32 for Cw7/YTS-2DL1; *n* = 34 for Cw7/YTS-2DL1 Y-27; *n* = 43 for Cw7/WASp KO). Statistical significance was calculated with one way ANOVA, and the data in the graph are represented as means ±SEM. (**F**). Representative images and the representative graph summarizing the relative pPLCγ2 (Y1217) synapse fluorescence intensities (*n* = 52 for Cw7/YTS-2DL1; *n* = 12 for Cw7/YTS-2DL1 Y-27; *n* = 21 for Cw7/WASp KO). Statistical significance was calculated with one way ANOVA, and the data in the graph are represented as means ± SEM.

Having demonstrated the importance of ARF velocity on SHP-1 conformational state, we next investigated the effect of WASp and myosin IIA on SHP-1 enzymatic activity using a Protein Tyrosine Phosphatase (PTP) assay in the presence or absence of Y-27, and in WASp KO cells. In this assay, the non-specific phosphatase substrate para-nitrophenylphosphate (PNPP) was used to detect SHP-1 activity. As expected, SHP-1 activity was markedly higher during inhibitory interactions (221-Cw4) relative to those that were activating (221-Cw7) (Figure 3A; 100% vs. 68.63 ± 2.27%, *p* ≤ 0.0006). However, the WASp KO cells demonstrated higher SHP-1 activity during activating interactions compared to WT cells (Figure 3A; 87.54 ± 5.22% vs. 68.63 ± 2.27%, *p* = 0.03).

Inhibition of myosin IIA (Y-27) during inhibitory interactions caused a reduction in SHP-1 activity to levels similar to those observed in activating interactions (Figure 3B; 71.49 ± 6.1% vs. 68.15 ± 5.5%, *p* = 0.99). These results indicate that the absence of WASp or myosin IIA not only leads to SHP-1 conformational change but also affects SHP-1 activity, suggesting that interference with the actomyosin network may slow the actomyosin flow and keep SHP-1 in active conformation or freeze the actomyosin flow and keep SHP-1 in its ‘closed’ conformation, maintaining the phosphorylation levels of signaling molecules. 

### 3.4. ARF Dynamics Regulate SHP-1 Catalytic Activity

Next, we determined the effect of ARF on the phosphorylation status/profile of the SHP-1 substrate, VAV1. VAV1 is a major protein required for NK cell activation which plays a role in activating small GTPase proteins of the Rho family, essential for actin cytoskeleton reorganization and lytic granule polarization towards target cells [44,45,46,47]. NK cell activation is also dependent on the rapid increase in intracellular calcium flux, which is crucial for NK cell effector functions, i.e., cytokine production and cytotoxicity [48,49]. The release of Ca^2+^ from the endoplasmic reticulum occurs following membrane recruitment and activation of PLCγ1/2 [50,51]. Following inhibitory receptor engagement, SHP-1 dephosphorylates PLCγ isoforms, resulting in NK cell inhibition [6]. Thus, to further examine the influence of actin dynamics on the abovementioned key signaling events in NK cells, we studied the effect of ARF modulation on VAV1 and PLCγ1/2 phosphorylation levels. To this end, YTS-2DL1 and 221-Cw4 or Cw7 cells were co-incubated at a 1:1 ratio and the conjugates were stained with antibody specifically recognizing the phosphorylated form of either VAV1 on tyrosine 160 (Y160) or PLCγ2 on tyrosine 1217 (Y1217). The cell conjugates were then analyzed to determine VAV1 or PLCγ2 activation status at the NKIS. As anticipated, almost no accumulation of either phosphorylated VAV1 or phosphorylated PLCγ2 was detected at the inhibitory NKIS in YTS-2DL1 or WASp KO cells, as determined by measuring pVAV1 (Y160) or pPLCγ2 (Y1217) synapse fluorescence intensities. Collectively the absence of WASp did not have a significant effect relative to the wild type (WT) in inhibitory interactions (221-Cw4) (Figure 3C,D, top and middle panels, *p* = 0.42, *p* = 0.89, respectively). Under these inhibitory conditions, suppression of F-actin dynamics using the myosin IIA inhibitor, Y-27, increased VAV1 and PLCγ2 phosphorylation levels in YTS-2DL1 cells (Figure 3C,D, top versus bottom panels, *p* < 0.0001, *p* = 0.04, respectively). We further examined the phosphorylation levels of VAV1 and PLCγ2 under activating interactions. Knockout of WASp resulted in a marked reduction in the phosphorylation levels of either VAV1 or PLCγ2 in comparison to WT cells at the activating NKIS (Figure 3E,F, top and middle panels, *p* < 0.0001, *p* = 0.0001, respectively). In contrast, following activating interactions, Y-27 treatment of WT cells did not lead to significant alterations in either VAV1 or PLCγ2 phosphorylation levels compared to untreated WT cells (Figure 3E,F, top and bottom panels, *p* = 0.31, *p* = 0.16, respectively). SHP-1 activity did not show significant alterations upon Y-27 treatment under activating conditions as indicated in Figure 3B. Collectively, these results demonstrate that ARF dynamics regulate SHP-1 activity, thereby influencing phosphorylation level of its substrates. 

### 3.5. The Role of WASp and Myosin IIA in the Regulation of the NK Cell Activating Response

**Figure 4:**
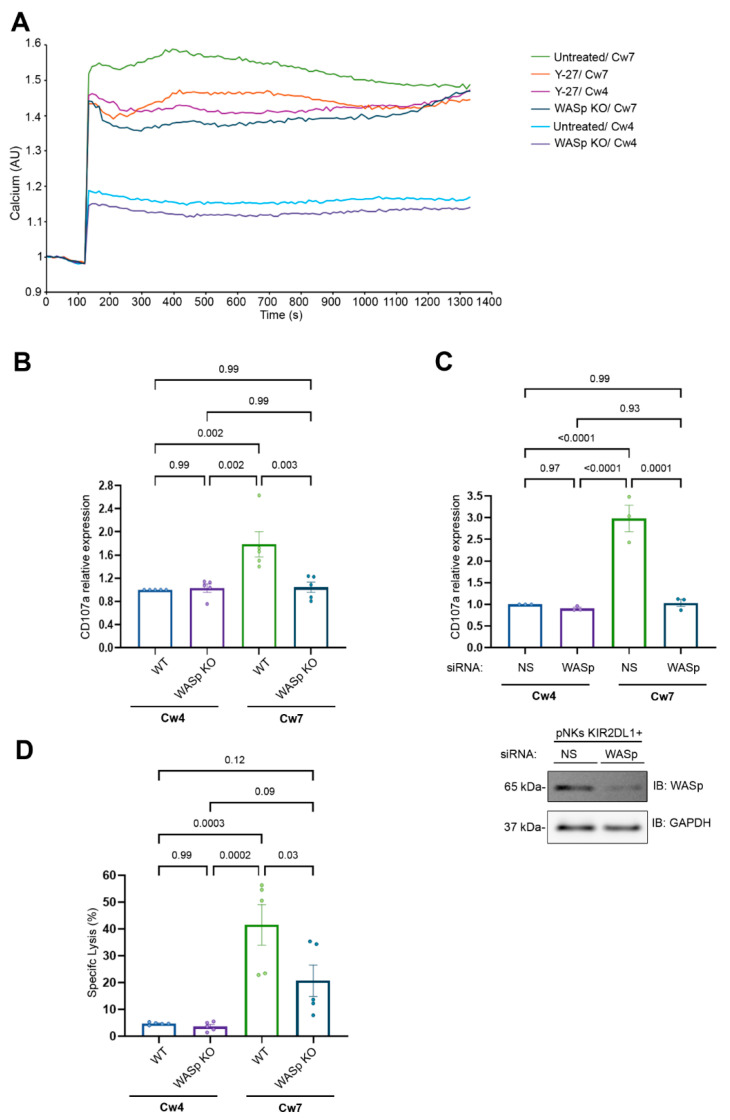
F-actin flow dynamics dictate the NK cell activation threshold. (**A**). YTS-2DL1, YTS-2DL1 treated with Y-27; WASp KO cells were loaded with Indo-1-AM and basal intracellular calcium levels were measured for 60 s. The target cells (221-Cw4 or 221-Cw7) were then added to the effector cells and incubated at 37 °C. Following 5-min incubation, the calcium levels were further analyzed by spectrofluorometer. A representative experiment of at least three independent experiments is shown. (**B**). YTS-2DL1 or WASp KO cells were incubated 221-Cw4 or 221-Cw7 target cells at a ratio of 1:2 for 2 h at 37 °C. Degranulation was measured by the expression of CD107a determined via FACS. Statistical significance was calculated with one way ANOVA test. Data are represented as means ±SEM of three independent experiments. (**C**). pNK KIR2DL1^+^ cells treated with either non-specific (NS) (control) or WASp siRNA were incubated 221-Cw4 or 221-Cw7 target cells at a ratio of 1:2 for 5 h at 37 °C. Degranulation was measured by the expression of CD107a measured by FACS. A representative blot demonstrating WASp silencing is shown (bottom). Statistical significance was calculated with one way ANOVA test. Data are represented as means ± SEM of three independent experiments. (**D**). YTS-2DL1 WT or WASp KO cells were incubated with [^35^S] Met-labeled 221-Cw4 or 221-Cw7 target cells at a ratio of 10:1 for 5 h at 37 °C. The specific lysis of target cells was measured as described in the Materials and Methods. Data are represented as means ± SEM of five independent experiments. Statistical significance was calculated with one way ANOVA.

We showed above that myosin IIA inhibition reduces SHP-1 activity at the inhibitory NKIS leading to increased PLCγ2 and VAV1 phosphorylation levels, and the absence of WASp increases SHP-1 activity following activating interactions, which results in reduced phosphorylation levels of these key signaling molecules. Next, the functional consequences of ARF suppression were further evaluated by measuring intracellular calcium flux. As expected, intracellular calcium levels following activating interactions (Figure 4A; 221-Cw7, green curve) were markedly higher relative to the levels observed following inhibitory interactions (Figure 4A; 221-Cw4, bright blue curve).

Strikingly, Y-27 treatment increased intracellular calcium levels following inhibitory interactions to levels comparable to those observed following activating interactions (Figure 4A; pink curve vs. green curve) but had minimal effect on the calcium level in the activating interaction (Figure 4A; orange versus green curve). Moreover, WASp KO cells exhibited reduced calcium flux levels in both the activating and inhibitory interactions (Figure 4A; dark blue curve vs. purple curve). These results clearly demonstrate that WASp plays a role in NK cell activation.

To examine the influence of the absence of the WASp on NK cell cytotoxicity, we measured the expression of CD107a, a marker of degranulating cytotoxic lymphocytes, on YTS-2DL1 WT vs. WASp KO cells (Figure 4B). We found a significant two-fold decrease in CD107a expression in YTS-2DL1 WASp KO cells during activating synapses, and no differences were observed upon inhibitory interactions. (WT/Cw7 vs. WASp KO/Cw7, *p* = 0.003; WT/Cw4 vs. WASp KO/Cw4, *p* = 0.99). Next, we verified the results with primary NK cells expressing KIR2DL1^+^ receptors (pNK KIR2DL1^+^) treated with WASp siRNA (Figure 4C) and incubated with target cells. As expected CD107a expression in WASp gene silenced pNK KIR2DL1^+^ was decreased in activating interactions compared to the pNK KIR2DL1^+^ treated with nonspecific (NS) siRNA (NS siRNA/Cw7 vs. WASp siRNA/Cw7, *p* = 0.0001; NS siRNA/Cw4 vs. WASp siRNA/Cw4, *p* = 0.97). The inability of WASp KO cells to degranulate following activating interactions (221-Cw7) suggests that WASp plays a role in the cytolysis of the target cells (Figure 4B,C). To confirm this, we performed target cell lysis via S^35^ Met assay. Indeed, the YTS-2DL1 WASp KO cells exhibited a significantly reduced ability to kill susceptible targets (221-Cw7), relative to the YTS-2DL1 WT (Figure 4D; 20.7 ± 5.86% vs. 41.57 ± 7.56%, *p* = 0.03). These results indicate that the loss of WASp indeed affects the NK cell cytotoxic response. Importantly, the NK cell activating response was suppressed to ultimately produce a response similar to that induced by inhibitory cells.

The requirement for myosin IIA in NK cell degranulation was previously well defined [52]. Our findings suggest that the ARF dynamics regulated by WASp play a central role in dictating the activation threshold and the functional outcome of NK cells.

## 4. Discussion

NK cells serve as the first line of defense of the innate immune system and are responsible for identifying and eradicating certain viral infections, other microbial diseases, parasites, and various types of cancer. NK cell activity is controlled by a large variety of inhibitory and activating receptors, and the balance between the signals determines whether the NK cell will kill its target or remain tolerant [53]. SHP-1 serves as a central regulator of the NK cell response, by both by responding to upstream signals, and by dephosphorylating proteins responsible for NK cell activation [54]. Though various studies demonstrated how SHP-1 regulates NK cell activation, very few have focused on the regulation of SHP-1 itself. We recently showed that ARF and Protein Kinase C (PKC)θ play an important role in determining the SHP-1 conformational structure and its impact on NK cell activity [9,33]. Along these lines, we also demonstrated that silencing of SHP-1 upon inhibitory synapse engagement improves NK cell cytotoxic potential [34]. In the current study, we demonstrate that the SHP-1-ARF axis is governed by WASp, the actin nucleation-promoting factor that dictates the NK cell immune response. 

Our previous findings demonstrate that the F actin dynamics and ARF at the NKIS site dictate the inhibitory and activating signals by regulating the SHP-1 conformational state and enzymatic activity, thereby modulating NK cell cytotoxicity [9]. Moreover, we suggested a mechanism whereby SHP-1 acts a mechanosensor, regulating NK cell activity. Accordingly, for SHP-1 to be activated, it must interact with the slow-moving actin at the inhibitory NKIS, where the pulling forces of actin exert tension on SHP-1, enabling the SH2 domain and catalytic domain to separate and allowing SHP-1 to acquire its open active conformation [9]. ARF inhibition, on the other hand, neutralizes the forces generated by actin movement, maintaining SHP-1 in its closed inactive conformation. As previously shown during activating interactions, SHP-1 is in a ‘closed’ conformational state mediated by fast ARF, and there is no binding of SHP-1 to β-actin. Thus, there is no interaction between the actomyosin forces that can ‘open’ SHP-1 and maintain it in its inactive conformation [9].

After revealing the significant role ARF plays in determining SHP-1 activity, and thus, NK cell activity, we show here that the lack of WASp significantly contributes to slow ARF movement. Following WASp knockout, ARF is decelerated, allowing SHP-1 to bind to actin, consequently decreasing NK cell activity. The above results demonstrate the existence of cross talk between the molecular and biophysical aspects of the cell, wherein cytoskeletal regulators can influence the conformational structure of SHP-1, a key phosphatase, and thereby regulate the phosphorylation levels of several downstream signal activators. In hematopoietic cells, the cytoskeleton is used as an anchor for various signaling molecules [55,56]. In fact, the actin cytoskeleton was regarded in the past as merely a static platform onto which signaling molecules could be anchored, and on which cytotoxic granules are immobilized before being secreted towards target cells [57]. The actin cytoskeleton is composed mainly of actin microfilaments and the motor protein, myosin IIA, forming the actomyosin network. Myosin IIA has an important role in two essential processes required for the NK cell effector response, generating the inward force for the centripetal actin movement and mediating the interaction of lytic granules with F-actin [58,59,60]. 

WASp is associated with a multi-protein complex together with myosin IIA at the NKIS. WASp has been extensively studied to date, including its role as a scaffold protein for assembly of effective signaling complexes, and its function as an important regulator for effective migration, phagocytosis and immune synapse formation in both myeloid and lymphoid immune cells [10,55]. In the current study, we reveal the role of WASp in modulating ARF velocity downstream to receptor engagement, thereby regulating SHP-1 conformational structure, and consequently, its enzymatic activity. These results suggest how the NK cell effector responses can be modulated by WASp activity in the TME. Indeed, this might explain why the NK cells of WAS patients demonstrate an impaired ability to secrete their cytolytic granules toward target cells and thus why these patients are susceptible to hematological malignancies [18,60]. 

WASp is a central hub in the signaling networks that control actin reorganization. When recruited to the cell membrane, WASp integrates a wide range of inputs from the cell’s microenvironment. In response, it activates the ubiquitous actin nucleating machine, the Arp2/3 complex. By controlling the degree, rate, and location of filament nucleation by Arp2/3, WASp proteins shape the structure and dynamics of filament networks. Here, we describe a novel outside-in signaling pathway mediated by WASp. Upon cellular activation, WASp triggers ARF and in turn regulates the enzymatic activity of SHP-1, a key phosphatase involved in dictating NK cell responsiveness. Furthermore, our data demonstrate that WASp KO cells exhibit reduced ARF, and they maintain the open conformational structure of SHP-1, thereby inactivating NK cell activity. This suggests that WASp contributes to fast ARF flow maintaining the closed (inactive) SHP-1 conformation, resulting in improved cytolytic potential. Additionally, WASp KO cells showed reduced degranulation and target lysis, similar to that seen in WAS patients, suggesting the potential of our studies to improve NK cell responsiveness by modulating various properties of the TME [42]. It is not yet clear if WASp directly controls granule trafficking and polarization or if this activity is mediated through ARF, which in turn signals the proteins responsible for effector responses. A recent study showed that WASp directly triggers actin patches, facilitating immune cell migration through dense tissues [61], an additional mechanosensing function. All together, these studies demonstrate the role of WASp in accelerating ARF velocity and thus as a molecular clutch in mechanosensitive scaffolds of the tumor microenvironment. Therefore, our results suggest that regulating the ARF may improve the NK cell response in the TME. 

Collectively, this study demonstrates the role of WASp as a regulator of the crosstalk between ARF and SHP-1. This interaction further impacts the molecular and biophysical mechanisms regulating the response of NK cells in the TME. Elucidating the mechanisms controlling NK cell activity will be helpful for understanding whether and how ex vivo perturbation of the NK cytoskeleton via regulatory molecules may hold promise for future therapeutic opportunities.

## 5. Conclusions

Our results open new avenues not only to explore the pathways controlling the normal NK cell response but also to understand the mechanisms regulating NK responsiveness under different pathologies, such as cancer or the primary immunodeficiencies WAS/XLT. We previously demonstrated the role of ARF in regulating NK activation through SHP-1; here, we show WASp and myosin IIA are additional players in this process. We demonstrate that ARF dynamics regulated by WASp and myosin IIA influence SHP-1, controlling its conformational structure and activity. Our research supports the notion of altering inhibitory to activating synapses and vice versa, through manipulation of SHP-1, thereby modulating NK cell cytotoxicity. Understanding the signaling cascades leading to the NK cellular response, and specifically the influence of ARF on SHP-1 activity, may lead to innovative approaches influencing NK cell functions in cancer, viral-infections, and autoimmune diseases.

## Data Availability

Data is contained within the article or supplementary material.

## Supplementary Materials

The following supporting information can be downloaded at: https://www.mdpi.com/article/10.3390/cancers14153756/s1, Video S1: WT, Video S2: WASp KO, Video S3: WT+Y27.
